# Employees’ Perceived Opportunities to Craft and In-Role Performance: The Mediating Role of Job Crafting and Work Engagement

**DOI:** 10.3389/fpsyg.2017.01876

**Published:** 2017-10-25

**Authors:** Jessica van Wingerden, Rob F. Poell

**Affiliations:** ^1^Centre of Research, Knowledge and Innovation, Schouten Global, Zaltbommel, Netherlands; ^2^Institute for Psychology, Faculty of Social Sciences, Erasmus University Rotterdam, Rotterdam, Netherlands; ^3^Department of Human Resource Studies, Tilburg University, Tilburg, Netherlands

**Keywords:** perceived opportunity to craft, job crafting, work engagement, in-role performance, JD-R theory

## Abstract

The present study was designed to gain knowledge of the relationship between employees’ perceived opportunities to craft, their actual job crafting behavior and, in line with JD-R theory, subsequently their work engagement and performance. Although scholars have suggested that employees’ perceived opportunities to craft their job may predict their actual job crafting behavior, which may have consequences for their well-being and performance, no study has examined the relationships between these variables. We collected data among a heterogeneous group of Dutch employees (*N* = 2090). Participants of the study reported their perceived opportunities to craft, job crafting behavior, work engagement and performance. Results indicated that individuals who experience a high level of opportunities to craft reported higher levels of job crafting behavior. In turn, perceived opportunities to craft and job crafting behavior related to higher levels of work engagement and subsequently performance. We discuss the implications of these findings for theory and practice.

## Introduction

The Fourth Industrial Revolution is emerging and has influenced jobs of employees all over the world, in a significant way. This impact may vary from job displacement to significant job creation, which has implications for employees’ knowledge, job skills, and behavior (World Economic Forum, 2017). Organizations that want to be responsive to change must constantly reinvent themselves and as a consequence facilitate employees’ reskilling. There is also a need for employees to be proactive and take their own responsibility to stay connected to their jobs and changing work environments. Employees can proactively optimize the fit between their (changing) job and their own talents, skills and interests by job crafting (Wrzesniewski and Dutton, 2001). Job crafting can be defined as employees’ self-initiated change behaviors that aim to align their jobs (and work environments) with their own preferences, motives, and passions (Wrzesniewski and Dutton, 2001; see also Berg et al., 2007). Since research revealed that job crafting behavior is positively related to employee’ well-being (Bakker et al., 2012) and work performance (Leana et al., 2009), organizations, senior management and researchers are interested in ways to stimulate job crafting behavior.

Literature suggests that employees’ actual job crafting behavior in the workplace may depend on their perceived opportunities to do so (Wrzesniewski and Dutton, 2001; Wrzesniewski, 2003; Van Wingerden et al., 2013; Van Wingerden, 2016; Van Wingerden and Niks, 2017). Insights in this proposed relation between employees’ job crafting perceptions and behavior may offer opportunities to organizations that want to create optimal conditions for employee well-being and performance. Therefore, the central aim of the present study is to examine the proposed relation between employees’ perceived opportunities to craft and their actual job crafting behavior, and in line with JD-R theory (Bakker and Demerouti, 2014), subsequently their work engagement and performance.

## Theoretical Background

### Job Crafting: Perception and Behavior

The concept of job crafting originates from 2001, when Wrzesniewski and Dutton (2001) labeled the self-initiated changes employees make to their jobs as “job crafting.” In the 15 years after publishing the article “Crafting a job: Revisioning employees as active crafters of their work” (Wrzesniewski and Dutton, 2001), job crafting has gained much interest among researchers all over the world. Employees’ proactive behavior at work was examined decades earlier. Back in the eighties and nineties, several studies already indicated that changes at work were not merely a top-down action by management but were also made and self-initiated by employees (Nicholson, 1984; Staw and Boettger, 1990). Wrzesniewski and Dutton (2001), coined job crafting as employees’ self-initiated, proactive behavior aimed at aligning their jobs with their own preferences, motives, and passions. Employees can craft their jobs by changing different aspects of their job like tasks, the relations at work and their cognitions about work (Wrzesniewski and Dutton, 2001).

According to LePine and Van Dyne (1998), these proactive changes that employees make in their job may result in permanent changes in their job design. Because job crafting involves initiating changes related to job characteristics and the design of the job, Tims et al. (2012) operationalized job crafting in line with Job Demands-Resources (JD-R) theory (Bakker and Demerouti, 2014). According to JD-R theory, every job consists of both job demands (aspects of the job that require energy) and resources (aspects of the job that give energy). Job demands are “the physical, social or organizational aspects of the job that require physical and/or cognitive engagement and that are associated with physical and psychological costs; job resources are those aspects of the job that help employees to achieve their work goals” (Demerouti et al., 2001, p. 501). Work pressure and complex assignments are examples of job demands; autonomy, opportunities for professional development and feedback examples of job resources. In line with the JD-R approach of job crafting, employees can craft their job by adapting their job demands and job resources. According to Tims et al. (2012), employees can craft their job by increasing their social job resources (e.g., seeking for colleague support), increasing structural job resources (e.g., enhancing one’s influence in decision making processes), increasing challenging job demands (e.g., initiating new projects) and decreasing hindering job demands (e.g., lowering the number of work tasks). However, earlier studies have shown ambiguous results of decreasing hindering job demands (Petrou et al., 2012; Tims et al., 2013; Van Wingerden et al., 2017b). Therefore, we will not include this fourth dimension in the current study.

There are similarities and differences between the two job crafting approaches by Wrzesniewski and Dutton (2001) and by Tims et al. (2012). Both job crafting approaches suggest that job crafting concerns employees’ self-initiated changes to optimize their work environments. The main difference between the two approaches is that Tims et al. (2012) define job crafting solely as observable employee’ behavior, whereas Wrzesniewski and Dutton (2001) explicitly include a cognitive element as well. According to Wrzesniewski and Dutton (2001), employees can alter their view of work. For example, service technicians working for internet providers may reframe their job as helping the world stay connected, as opposed to merely maintaining the digital infrastructure. By changing the way employees look at their work, they may experience their work in a more positive way. By crafting their job, employees can optimize both their work environment and their work experience.

Whether or not employees will proactively craft their job, may depend on internal and external factors (Wrzesniewski and Dutton, 2001; Wrzesniewski, 2003; Van Wingerden and Niks, 2017). For example, employees with a proactive personality or who are self-efficacious (both internal factors) may proactively craft their job because they feel they are able to do so. Further, employees who experience they have a sufficient level of decision-making freedom and feel autonomous at work or who have opportunities to craft their job (both external factors) may be more likely to optimize their work environment because they feel supported to proactively take charge at work themselves. Different studies revealed the proposed positive relationships between internal factors like proactive personality and approach temperament and job crafting behavior (Bakker et al., 2012; Bipp and Demerouti, 2015), and between external factors like job resources and leadership and job crafting behavior (Petrou et al., 2012; Gordon et al., 2015). This study is one of the first that examined the relationship between employees’ opportunities to craft (an external factor) and their job crafting behavior. Perceived opportunity to craft can be defined as employees’ perceptions regarding their opportunity to craft their jobs (Van Wingerden and Niks, 2017). We suggest to expand this definition of perceived opportunity to craft by including the description of job crafting behavior based on the similarities between the two job crafting approaches by Wrzesniewski and Dutton (2001) and by Tims et al. (2012). In line with this perspective, perceived opportunity to craft can be defined as employees’ perceptions regarding their opportunities to proactively optimize their work environment. Employees’ perceived opportunity to craft may be influenced by management behavior. For example, managers who give their employees autonomy in their job and (positive) feedback on their job crafting actions may positively affect their perceived opportunities to craft (see also Wrzesniewski, 2003). If we take a closer look at this example, employees’ perceived opportunities to craft may be predicted by job resources like autonomy and feedback. Thus, employees’ perceived opportunities to craft may also be influenced by characteristics of their work environment. This idea is in line with the findings of a study by Van Wingerden and Niks (2017), which revealed that perceived opportunities to craft and job resources (autonomy and opportunities for professional development) are positively related, yet distinctive constructs. The perception of (not) having opportunities to craft by itself may also directly affect work attitudes (Van Wingerden and Niks, 2017).

A qualitative study among teachers who participated in a job crafting training (Van Wingerden et al., 2013) confirmed the assumption that employees’ perceived opportunities to craft may determine whether they will craft their jobs. Teachers who participated in the job crafting training and reported they did *not* succeed crafting their job stated that they did not perceive opportunities to do so. These employees felt that making changes in their work environment was restricted by managers, behavioral patterns on the job, and the organization. In contrast, colleagues who reported they successfully made changes to their work environments perceived they had opportunities to craft their job. In line with these findings, we hypothesize:

Hypothesis 1: Employees’ perceived opportunities to craft are positively related to employees’ job crafting behavior.

### Relationships between Job Crafting, Work Engagement, and Performance

Employees who engage in job crafting proactively work on congruence between their talents, strengths and interests and the changing work environment. By doing so, employees may stay challenged in their job, and maintain their level of joy and energy at the same time. In other words, when crafting their job employees may feel engaged at work. Employees’ who are engaged at work, are having a sense of energetic and effective connection with their job and feel they are able to deal with their job demands. Schaufeli and Bakker (2004, p. 295), defined work engagement as “the positive, fulfilling and work-related state of mind that is characterized by vigor, dedication, and absorption.” Employees who are vigorous, are experiencing high levels of energy and mental resilience at work. Employees’ dedication refers to the involvement in their job while experiencing a sense of enthusiasm and significance. Employees’ absorption refers to being fully concentrated and immersed in their work (Schaufeli and Bakker, 2010). Thus, engaged individuals often forget about time as it flies by when they are at work. Research revealed that employees who are engaged at work are healthier than their less-engaged colleagues and experience more positive emotions (Bakker and Demerouti, 2014).

Work engagement can be predicted using the JD-R model (Bakker and Demerouti, 2014). The JD-R model provides a clear overview of the ways in which demands, resources, psychological states, and outcomes are associated. According to Bakker and Demerouti (2007), every job consists of two job characteristics; job demands and (job) resources. The JD-R model states that the combination of high job demands and high resources leads to high levels of motivation, involvement, and work engagement (Tuckey et al., 2012), which in turn leads to high levels of performance (Bakker and Bal, 2010; Bakker and Demerouti, 2014; Van Wingerden et al., 2015). Employees may increase their own work engagement and performance through job crafting by optimizing their job demands and resources. Several survey, diary and intervention studies have revealed a positive relationship between job crafting and work engagement (e.g., Bakker et al., 2012; Petrou et al., 2012; Van Wingerden et al., 2017a). More specific, job crafting behavior predicts work engagement when employees focus on increasing their challenging job demands and their resources (Van Wingerden et al., 2017b). These findings underline the focus of the current study on job crafting behaviors related to increasing challenges and resources. Research has revealed that the relationship between job crafting and work engagement is dynamic; job crafting may be a cause of being engaged at work (Van Wingerden et al., 2017a) but also a consequence (Fritz and Sonnentag, 2009; Schaufeli et al., 2009; Parker et al., 2010). This is consistent with the Job Demands-Resources model within JD-R theory (Bakker and Demerouti, 2014), which includes a feedback loop from outcomes of work engagement to antecedents of work engagement.

Employees who craft their job are highly valuable for organizations; they are engaged and realize their own and organizational goals. Employees who realize their work goals are expected to score high on in-role performance. According to Motowidlo and Van Scotter (1994), in-role performance comprises those required outcomes and employee behaviors as described in employees’ job profile, which contribute to the goals of the organization. Different studies have indeed confirmed the proposed positive relations between job crafting on the one hand, and individual and organizational outcomes on the other (Leana et al., 2009; Tims et al., 2013; Van Wingerden, 2016; Van Wingerden et al., 2017b). A study among 95 dyads of employees showed that employees’ job crafting behavior was predictive of their work engagement and colleague-ratings of in-role performance (Bakker et al., 2012). Further, a longitudinal job crafting study (Tims et al., 2016) among 288 participants showed similar positive relations between employees’ job crafting behavior and their work engagement and subsequently their performance. These studies indicate that employees who take the initiative themselves to optimize their job demands and job resources in the work environment, facilitate and stimulate their own work engagement and subsequently their performance. We therefore hypothesize:

Hypothesis 2: Employees’ job crafting behavior is positively related to employees’ work engagement.

Hypothesis 3: Employees’ work engagement is positively related to employees’ in-role performance.

In addition to the proposed positive relations between perceived opportunities to craft and job crafting behavior, between job crafting behavior and work engagement, and between work engagement and performance, our theoretical arguments suggest that perceived opportunities to craft influence performance through job crafting behavior and work engagement. Employees who perceive opportunities to craft their job may proactively optimize their job demands and resources to align their working conditions to their own needs and abilities. By optimizing their working conditions via job crafting, employees create a work environment that fosters their enthusiasm and engagement and subsequently their performance. In line with Wrzesniewski and Dutton (2001), we hypothesize that the relation between perceived opportunities to craft and work engagement is mediated by employees’ job crafting behavior. In addition, following JD-R theory (Bakker and Demerouti, 2014), Van Wingerden and Niks (2017), we propose that job crafting behavior can explain the association between perceived opportunities to craft and work engagement, and accordingly performance. Thus, we hypothesize:

Hypothesis 4: Perceived opportunity to craft has a positive relationship with in-role performance, through first job crafting and then work engagement (sequential mediation).

## Materials and Methods

### Participants and Procedure

We collected data using an online survey. The study was announced on a well-known Dutch career development website as well as through various social media channels. Data was collected from a diverse population to increase the heterogeneity of the participants, which facilitates generalization of the research findings (Demerouti and Rispens, 2014). Respondents were invited to participate on a voluntary basis and directed to the survey through an online link. The survey was in Dutch and available for 3 weeks. In total, 2061 employees filled out the survey. A majority of the sample was female (55.2%) and the mean age of the participants was 47.5 years (*SD* = 9.3). Most participants (77.2%) reported to possess at least a bachelor’s degree. Various sectors were represented, with participants working in health care (20.0%), education (15.4%), professional services (15.2%), the public sector (14.1%), industry (13.8%), information technology (11.2%), and financial services (10.3%). Data was collected in accordance with the ethical guidelines of the Dutch Association of Psychologists and also the American Psychological Association and the. In line with the ethical guidelines, participation was completely voluntary, data collection through a self-report survey is exempted from an institutional ethics committee’s approval, and the respondents did not receive any compensation for their contribution. Informed consent was given by clicking on the “Finish” button at the end of the survey.

### Measures

*Perceived opportunity to craft* was measured with the five-item scale developed by Van Wingerden and Niks (2017). An example is: “At work I have the opportunity to vary the type of tasks I carry out.” Participants had to score the items on a 7-point scale ranging from 1 (totally disagree) to 7 (totally agree).

*Job crafting* was measured using 3 subscales of the job crafting questionnaire developed by Tims et al. (2012). Of each subscale, four items were included and scored on a five-point scale ranging from 1 (*never*) to 5 (*very often*). Examples are: “I ask colleagues for advice” (increasing social job resources), “I regularly take on extra tasks even though I do not receive extra salary for them” (increasing challenging job demands), and “I try to learn new things at work” (increasing structural job resources).

*Work engagement* was measured with the Utrecht Work Engagement Scale (UWES; Schaufeli et al., 2006). The instrument consists of nine items and three subscales to assess vigor, dedication, and absorption. Examples for each subscale are “At work, I am bursting with energy” (vigor), “I am enthusiastic about my job” (dedication), and “I am immersed in my work” (absorption). Participants could respond to these items using a 7-point frequency scale ranging from 0 (*never*) to 6 (*always*).

*In-role Performance* was measured using the In-role Performance scale (Williams and Anderson, 1991), which consists of seven items. A sample item is: “I adequately complete all of my assigned duties.” Participants had to score the items on a five point scale ranging from (1) totally disagree to (5) totally agree.

### Analysis

**FIGURE 1:**
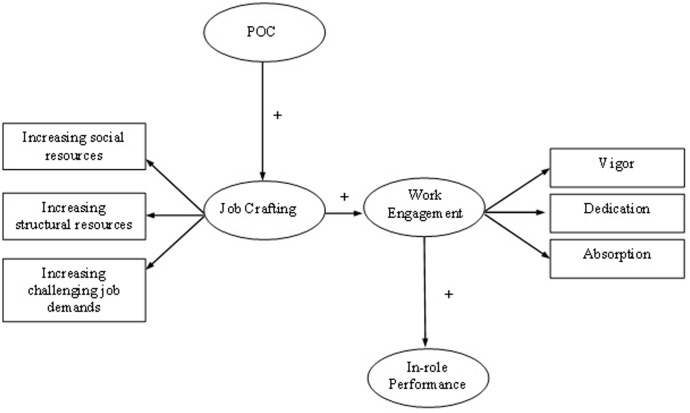
Job crafting perception and behavior model.

The job crafting perception and behavior model (see **Figure 1**) was tested with structural equation modeling (SEM) analyses using the AMOS software package (Arbuckle, 2005). To test the fit of the, measurement model, and alternative models to the data, the traditional chi-square, the goodness-of-fit index (GFI), and the root mean square error of approximation (RMSEA) were tested. As a rule of thumb, a GFI > 0.90 and RMSEA < 0.08 indicate a reasonable fit of the model to the data (Browne and Cudeck, 1993). As recommended by Marsh et al. (1996), the incremental fit index (IFI), and the comparative fit index (CFI) were also assessed. These values should meet the criterion of 0.90 (Hoyle, 1995). Little et al. (2002), revealed that using parcels in testing structural equation modeling result in more reliable measurement models. We therefore conducted our SEM analysis on a partial disaggregation model (Bagozzi and Edwards, 1998) by creating parcels of items as also recommended by Hall et al. (1999).

We created parcels of items for the variables ‘Perceived opportunity to craft’ and ‘In-role performance,’ which were both included in the model as latent factors with two indicators. ‘Job crafting’ and ‘Work engagement’ were included as latent factors with their abovementioned subscales as the indicators. We examined whether significant pathways between perceived opportunity to craft and in-role performance represented indirect relationships by means of bootstrapping.

## Results

### Descriptive Statistics

The means, standard deviations, reliabilities, and correlations between all study variables are displayed in **Table 1**. In order to test the construct validity of the scale variables perceived opportunity to craft, job crafting, work engagement, and in-role performance, we tested a measurement model with the parcels tapping these latent variables. This measurement model showed an adequate fit to the data: χ2(29) = 167.324, RMSEA = 0.048, GFI = 0.984, NNFI = 0.980, IFI = 0.987, CFI = 0.987. All parcels had significant loadings on the intended factors (range λ = 0.40-0.96; *p* < 0.001).

**Table 1:** Means, standard deviations, correlations, and cronbach’s alpha coefficients (on the diagonal) of the research variables.

		*M*	*SD*	1	2	3	4	5	6	7	8
(1)	Perceived opportunity to craft	5.02	1.07	(0.86)							
(2)	JC: Increasing structural job resources	4.31	0.53	0.41^∗∗^	(0.76)						
(3)	JC: Increasing social job resources	3.88	0.65	0.29^∗∗^	0.33^∗∗^	(0.79)					
(4)	JC: Increasing challenging demands	3.14	0.69	0.22^∗∗^	0.51^∗∗^	0.27^∗∗^	(0.71)				
(5)	Vigor	4.99	1.08	0.46^∗∗^	0.45^∗∗^	0.20^∗∗^	0.38^∗∗^	(0.87)			
(6)	Dedication	5.25	1.13	0.48^∗∗^	0.45^∗∗^	0.21^∗∗^	0.34^∗∗^	0.84^∗∗^	(0.90)		
(7)	Absorption	4.81	1.02	0.30^∗∗^	0.35^∗∗^	0.15^∗∗^	0.35^∗∗^	0.67^∗∗^	0.67^∗∗^	(0.75)	
(8)	In-role performance	4.21	1.04	0.21^∗∗^	0.38^∗∗^	0.11^∗∗^	0.34^∗∗^	0.38^∗∗^	0.34^∗∗^	0.29^∗∗^	(0.89)

*^∗∗^p < 0.01; JC, job crafting.*

### Hypotheses Testing

**FIGURE 2:**
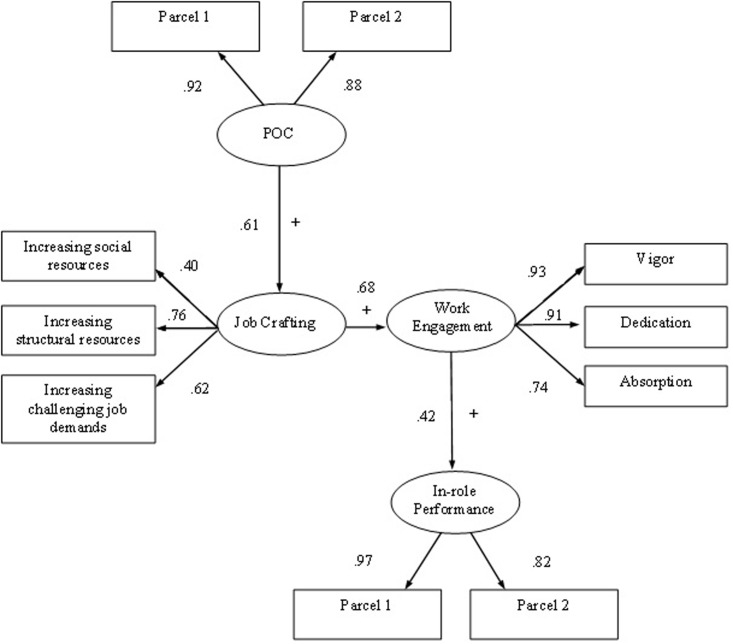
Maximum likelihood estimates for the job crafting perception and behavior model. *N* = 2190. All factor loadings and path coefficients are significant at the *p* < 0.001 level.

Our central prediction is that employees’ perceived opportunity to craft is positively related to employees’ job crafting behavior (H1), and in turn employees’ job crafting behavior is positively related to their work engagement (H2), subsequently work engagement is positively related to employees’ in-role performance (H3). In addition, we stated that perceived opportunity to craft has a positive relationship with in-role performance, through first job crafting and then work engagement (sequential mediation) (H4). To test these hypotheses, we conducted SEM analyses. The results of the SEM analyses indicated that the hypothesized model fit well to the data: χ2(32) = 409.876, RMSEA = 0.076, GFI = 0.960, NNFI = 0.952, IFI = 0.966, CFI = 0.966. Results showed that perceived opportunity to craft was positively related to job crafting (β = 0.61, *p* < 0.001; see also **Figure 2**). Job crafting, in turn, was a significant predictor of work engagement (β = 0.68, *p* < 0.001). Finally, work engagement was significantly related to in-role performance (β = 0.42, *p* < 0.001). These findings offer evidence for Hypotheses 1, 2, and 3. As literature has revealed that work engagement may also be a predictor of how employees perceive their work environment and a predictor of proactive behavior (Parker and Griffin, 2011), we tested a theoretically plausible alternative model. We built a new model in which work engagement predicted first perceived opportunities to craft, and then job crafting and performance. This alternative model did not, however, yield an acceptable fit to the data: χ2(32) = 517.443, RMSEA = 0.087, GFI = 0.953, NNFI = 0.938, IFI = 0.956, CFI = 0.956. The RMSEA above 0.08 indicates there is no reasonable fit of the model (Browne and Cudeck, 1993).

In additional series of SEM analyses, we tested two alternative models, namely the partial mediation model and the direct effects model. In addition to the sequential mediation of the proposed model, the partial mediation model also includes a direct relationship between perceived opportunity to craft and in-role performance. This alternative model also showed acceptable fit to the data: χ2(31) = 407.376, RMSEA = 0.077, GFI = 0.960, NNFI = 0.950, IFI = 0.966, CFI = 0.966. In addition, the proposed model did not fit significantly better to the data than the partial mediation model: Δχ2(1) = 2.500, *p* = 0.114. Since both models had similar fit indices, the most preferred model would be the one with the least complexity. In order to test which model was the least complex, we calculated Akaike’s Information Criterion (AIC) for each of the models, whereby a lower value is an indication of better fit. The proposed model had a slightly lower AIC value (AIC = 455.376) compared to the partial mediation model (AIC = 455.876). In addition to the sequential mediation of the proposed model, the direct effects model includes only the direct relationships between perceived opportunities to craft, job crafting, and work engagement on the one hand, and in-role performance on the other. The direct effects model showed a bad fit to the data: χ^2^(31) = 621.901, RMSEA = 0.096, GFI = 0.947, NNFI = 0.922, IFI = 0.946, CFI = 0.946. The proposed model fit significantly better to the data than the direct effects model: Δχ^2^ (1) = 212.025, *p* < 0.001. These results offer additional evidence for the proposed model.

According to Hypothesis 4, perceived opportunity to craft is positively related to in-role performance, through job crafting and work engagement. These indirect effects were examined using the bootstrap analysis option in AMOS (MacKinnon, 2008). Specifically, we tested three indirect effects using bootstrapping with 95% confidence intervals. First, we tested the indirect effect of perceived opportunity to craft on work engagement through job crafting. The results of the bootstrap analysis showed that this indirect effect was significant (β = 0.413, *p* = 0.025). The bias corrected confidence interval (B-CCI) ranged from 0.357 to 0.451. Second, we tested the indirect effect of job crafting on in-role performance through work engagement. This indirect effect was also significant (β = 0.285, *p* = 0.007, 0.244 ≤ B-CCI ≤ 0.326). The results of the third bootstrap analysis showed that the sequential mediation effect was significant as well (β = 0.173, *p* = 0.007, 0.140 ≤ B-CCI ≤ 0.202). These findings offer support for our hypothesized sequential mediation effect from perceived opportunity to craft to in-role performance through job crafting and work engagement.

## Discussion

In this paper, we argued that employees’ actual job crafting behavior that leads to well-being and performance may depend on employees’ perceived opportunities to craft their jobs (Van Wingerden and Niks, 2017). We hypothesized that employees who perceive they have the opportunity to craft their jobs would be most likely to optimize job demands and resources in their work environments through job crafting. As a consequence, these employees become engaged at work (Bakker et al., 2012) and perform well (Leana et al., 2009). The results of our study were consistent with the hypotheses. Employees’ perceived opportunities to craft had a positive relationship with their in-role performance through job crafting behavior and work engagement. In the next section, we discuss the contributions of our study.

### Theoretical Contributions

A first contribution of this article is that it offers evidence for a positive relation between employees’ perceived opportunities to craft and their job crafting behavior. Literature suggests that employees’ actual job crafting behavior may depend on their perceived opportunities to craft their jobs (Wrzesniewski and Dutton, 2001; Wrzesniewski, 2003; Van Wingerden et al., 2013; Van Wingerden and Niks, 2017), however, until now no study had empirically examined this proposed relation among employees in several occupational groups. Therefore, this study may help researchers to gain more insight into the relations between job crafting perception and behaviors, as well as its consequences. In earlier studies it was stated that actual job crafting behavior may depend on internal and external factors (Wrzesniewski and Dutton, 2001; Wrzesniewski, 2003; Van Wingerden and Niks, 2017). In line with this idea, job crafting has indeed been shown to be positively related with approach temperament (Bipp and Demerouti, 2015) and proactive personality (Bakker et al., 2012) (both internal factors), and with job resources and leadership (Petrou et al., 2012; Gordon et al., 2015) (both external factors). This study is one of the first that confirmed that opportunities to craft, which is an external factor, is positively related to job crafting behavior. The boundary conditions for the relationship between employees’ perceived opportunities to craft and their job crafting behavior need to be further explored. The extent to which employees who perceive opportunities to craft will actually craft their job may be influenced by different factors. For instance, employees who are self-efficacious and have confidence in their own ability to achieve intended results may feel able to participate in new projects at work and will actually do so. In line with this example, personal resources (internal factors) like self-efficacy may even moderate the relationship between perceived opportunities to craft and job crafting behavior. In a similar vein, employees who experience they have a sufficient level of decision-making freedom and are autonomous at work (external factors) may perceive they have opportunities to craft their job and will proactively optimize their work environment. Thus, job resources like autonomy may also predict, mediate or moderate the relationship between perceived opportunities to craft and job crafting behavior. Future research may further examine these complex and dynamic relationships between employees, their behaviors and work contexts.

Second, employees who perceive they have opportunities to craft their job proactively align their job demands and resources with their own abilities and needs. By doing so they take their own responsibility to stay connected to their changing work environments. This may not only facilitate work engagement and, indirectly, performance, but also contribute to their sustainable employability. Our study is among the first to show the importance of perceived opportunity to job craft in employees’ work engagement and, ultimately, their performance.

### Limitations and Suggestions for Future Research

Although this study provides evidence for the proposed model and hypotheses, some limitations of our study need to be acknowledged. One limitation is the self-report nature of our data. By using self-reports we cannot entirely avoid the risk of common method bias, which may inflate the correlations between the variables. However, employees’ evaluations of the job and their well-being may be subjective, and self-reports may be the best method to capture these perceptions and feelings (Sousa-Poza and Sousa-Poza, 2000). To diminish potential common method bias (Podsakoff et al., 2003) it may be interesting to include different types of measures such as peer ratings of job crafting behavior and work engagement (cf. Tims et al., 2012). In addition, future studies may also pay attention to other ratings provided by supervisors, or clients, for example for measuring in-role performance. This will give a more objective view in terms of observable behavior.

Second, a consequence of the cross-sectional nature of our data is that we cannot warrant causality in our study design since predictor, mediators, and outcome variables are not temporally separated. Future research should try to replicate our study using a longitudinal design to examine the causal relationships among the study variables. Third, the present studies included one heterogeneous convenience sample. A strength is that the sample included employees from a variety of industries (e.g., health care, education, and professional services). Nevertheless, all participants were Dutch employees, which may limit the generalizability of our findings. Future research may further add to the generalizability of the findings by replicating our study among employees in different countries and cultures. Fourth, this study specifically focused on perceived opportunities to craft and its outcomes. Future studies should also examine the antecedents of perceived opportunities to craft. These insights may further support professionals and organizations that want to facilitate employees’ job crafting behavior. Despite these limitations, the results of this study indicate that the concept of perceived opportunity to craft is useful for studying how employees’ perceptions and behaviors contribute to their well-being and organizational outcomes.

### Practical Implications and Conclusion

Translating the findings of this study into practical implications we suggest that employees’ perceived opportunities to craft their jobs may determine their actual job crafting behavior in the workplace. Managers should be aware of the importance of these perceptions, as they wish to stimulate employees’ job crafting behaviors. For example, they can provide room and opportunities for employees to engage in job crafting and appreciate and share positive job crafting experiences of colleagues. By doing so, managers underline that they support employees’ job crafting behaviors, which may positively influence employees’ perceived opportunities to craft. In addition, they can facilitate a dialog on the added value of job crafting behavior in the workplace or stimulate job crafting through training (Van Wingerden et al., 2017b). Evidence from job crafting interventions has shown that job crafting behaviors can be stimulated trough training (Van Wingerden et al., 2013, 2017a; Gordon, 2015; Van den Heuvel et al., 2015), and enhanced job crafting behavior after a job crafting training seems to be sustainable over time (Van Wingerden et al., 2017b). Managers who positively influence employees’ perceived opportunities to craft before offering job crafting interventions in the organization can create optimal conditions that may in fact strengthen intervention effects. Future research may test this proposed positive relationship between perceived opportunity to craft and job crafting intervention effects.

For employees, this study underlines the importance of taking charge of the congruence between their changing work environments and their own preferences, motives, and passions. In changing work environments, job profiles are more and more about responsibilities and results and less about predefined tasks. In line with this focus, organizations expect employees to be self-directed and take responsibility for their in-role performance. Proactively optimizing the work environment therefore seems to be a beneficial strategy to stay connected to the job.

## Conclusion

This study has shed a light on the unique role of perceived opportunities to craft. It demonstrates that opportunities to craft may operate as a driver for employees’ actual job crafting behavior that is positive related to work engagement and, subsequently, performance. We strongly believe in the potential of employees’ proactive behavior and the merits that the cultivation of this proactive behavior has for employees and organizations. Therefore, we hope this study will invite other researchers to further explore the role of perceived opportunities to craft beyond this study. Ultimately we hope this study will inspire practitioners to actively cultivate employees ‘opportunities to craft and therewith job crafting behavior within their organizations.

## Author Contributions

JvW designed the study, collected the data and analyzed the data. JvW and RP both drafted the manuscript.

## Conflict of Interest Statement

The authors declare that the research was conducted in the absence of any commercial or financial relationships that could be construed as a potential conflict of interest.

## References

[B1] Arbuckle J. L. (2005). *Amos 6.0 User’s Guide.* Chicago, IL: SPSS, Inc.

[B2] Bagozzi R. P. Edwards J. R. (1998). A general approach for representing constructs in organizational research. *Organ. Res. Methods* 1 45–87. 10.1177/109442819800100104

[B3] Bakker A. B. Bal P. M. (2010). Weekly work engagement and performance: a study among starting teachers. *J. Occup. Organ. Psychol.* 83 189–206. 10.1348/096317909X402596

[B4] Bakker A. B. Demerouti E. (2007). The Job Demands-Resources model: state of the art. *J. Manag. Psychol.* 22 309–328. 10.1108/02683940710733115

[B5] Bakker A. B. Demerouti E. (2014). “Job demands-resources theory,” in *Wellbeing: A Complete Reference Guide*, eds Cooper C. Chen P. (Chichester: Wiley-Blackwell), 37–64.

[B6] Bakker A. B. Tims M. Derks D. (2012). Proactive personality and job performance: the role of job crafting and work engagement. *Hum. Relat.* 65 1359–1378. 10.1177/0018726712453471

[B7] Berg J. M. Dutton J. E. Wrzesniewski A. (2007). *What is Job Crafting and Why Does It Matter.* Available at: http://positiveorgs.bus.umich.edu/wp-content/uploads/What-is-Job-Crafting-and-Why-Does-it-Matter1.pdf [accessed, March 3, 2016].

[B8] Bipp T. Demerouti E. (2015). Which employees craft their jobs and how? Basic dimensions of personality and employees’ job crafting behaviour. *J. Occup. Organ. Psychol.* 88 631–655. 10.1111/joop.12089

[B9] Browne M. W. Cudeck R. (1993). “Alternative ways of assessing model fit,” in *Testing Structural Equation Models*, eds Bollen K. A. Long J. S. (Newbury Park, CA: Sage), 445–455.

[B10] Demerouti E. Bakker A. B. De Jonge J. Janssen P. P. M. Schaufeli W. B. (2001). Burnout and engagement at work as a function of demands and control. *Scand. J. Work Environ. Health* 27 279–286. 10.5271/sjweh.615 11560342

[B11] Demerouti E. Rispens S. (2014). Improving the image of student-recruited samples: a commentary. *J. Occup. Organ. Psychol.* 87 34–41. 10.1111/joop.12048

[B12] Fritz C. Sonnentag S. (2009). Antecedents of day-level proactive behavior: a look at job stressors and positive affect during the workday. *J. Manage.* 35 94–111. 10.1177/0149206307308911

[B13] Gordon H. J. (2015). *Craft Your Job!: Improving Well-Being, Decision Making, and Performance in Healthcare.* Doctoral dissertation, University of Technology, Eindhoven.

[B14] Gordon H. J. Demerouti E. Le Blanc P. M. Bipp T. (2015). Job crafting and performance of Dutch and American health care professionals. *J. Pers. Psychol.* 14 192–202. 10.1027/1866-5888/a000138

[B15] Hall R. J. Snell A. F. Foust M. (1999). Item parceling strategies in SEM: investigating the subtle effects of unmodeled secondary constructs. *Organ. Res. Methods* 2 233–256. 10.1177/109442819923002

[B16] Hoyle R. H. (1995). *Structural Equation Modeling: Concepts, Issues, and Applications.* Thousand Oaks, CA: SAGE.

[B17] Leana C. Appelbaum E. Shevchuk I. (2009). Work process and quality of care in early childhood education: the role of job crafting. *Acad. Manage. J.* 52 1169–1192. 10.5465/AMJ.2009.47084651

[B18] LePine J. A. Van Dyne L. (1998). Predicting voice behavior in work groups. *J. Appl. Psychol.* 83 853–868. 10.1037/0021-9010.83.6.853

[B19] Little T. D. Cunningham W. A. Shahar G. Widaman K. F. (2002). To parcel or not to parcel: exploring the question, weighing the merits. *Struct. Equ. Model.* 9 151–173. 10.1207/S15328007SEM0902_1

[B20] MacKinnon D. P. (2008). *Introduction to Statistical Mediation Analysis.* New York, NY: Routledge.

[B21] Marsh H. W. Balla J. R. Hau K. T. (1996). “An evaluation of incremental fit indices: a clarification of mathematical and empirical properties,” in *Advanced Structural Equation Modeling: Issues and Techniques*, eds Marcoulides G. A. Schumacker R. E. (Mahwah, NJ: Lawrence Erlbaum Associates), 315–353.

[B22] Motowidlo S. J. Van Scotter J. R. (1994). Evidence that task performance should be distinguished from contextual performance. *J. Appl. Psychol.* 79 475–480. 10.1037/0021.9010.79.4.475 10948797

[B23] Nicholson N. (1984). A theory of work role transitions. *Adm. Sci. Q.* 29 172–191. 10.2307/2393172

[B24] Parker S. K. Bindl U. Strauss K. (2010). Making things happen: a model of proactive motivation. *J. Manage.* 36 827–856. 10.1177/0149206310363732 23379914

[B25] Parker S. K. Griffin M. A. (2011). Understanding active psychological states: embedding engagement in a wider nomological net and closer attention to performance. *Eur. J. Work Organ. Psychol.* 20 60–67. 10.1080/1359432X.2010.532869

[B26] Petrou P. Demerouti E. Peeters M. C. W. Schaufeli W. B. Hetland J. (2012). Crafting a job on a daily basis: contextual correlates and the link to work engagement. *J. Organ. Behav.* 33 1120–1141. 10.1002/job.1783

[B27] Podsakoff P. M. MacKenzie S. B. Lee J. Podsakoff N. P. (2003). Common method bias in behavioural research: a critical review of the literature and recommended remedies. *J. Appl. Psychol.* 88 879–903. 10.1037/0021-9010.88.5.879 14516251

[B28] Schaufeli W. B. Bakker A. B. (2004). Job demands, job resources and their relationship with burnout and engagement: a multi sample study. *J. Organ. Behav.* 25 293–315. 10.1002/job.248

[B29] Schaufeli W. B. Bakker A. B. (2010). “The conceptualization and measurement of work engagement: a review,” in *Work Engagement: A Handbook of Essential Theory and Research*, eds Bakker A. B. Leiter M. P. (New York, NY: Psychology Press).

[B30] Schaufeli W. B. Bakker A. B. Salanova M. (2006). The measurement of work engagement with a short questionnaire: a cross-national study. *Educ. Psychol. Meas.* 66 701–716. 10.1177/0013164405282471

[B31] Schaufeli W. B. Bakker A. B. Van Rhenen W. (2009). How changes in job demands and resources predict burnout, work engagement, and sickness absenteeism. *J. Organ. Behav.* 30 893–917. 10.1002/job.595

[B32] Sousa-Poza A. Sousa-Poza A. A. (2000). Well-being at work: a cross-national analysis of the levels and determinants of job satisfaction. *J. Socioecon.* 29 517–538. 10.1016/S1053-5357(00)00085-8

[B33] Staw B. M. Boettger R. D. (1990). Task revision: a neglected form of work performance. *Acad. Manage. J.* 33 534–559. 10.2307/256580

[B34] Tims M. Bakker A. B. Derks D. (2012). Development and validation of the job crafting scale. *J. Vocat. Behav.* 80 173–186. 10.1016/j.jvb.2011.05.009

[B35] Tims M. Bakker A. B. Derks D. (2016). Job crafting and its relationships with person–job fit and meaningfulness: a three-wave study. *J. Vocat. Behav.* 92 44–53. 10.1016/j.jvb.2015.11.007

[B36] Tims M. Bakker A. B. Derks D. Van Rhenen W. (2013). Job crafting at the team and individual level: implications for work engagement and performance. *Group Organ. Manage.* 38 427–454. 10.1177/1059601113492421

[B37] Tuckey M. R. Bakker A. B. Dollard M. F. (2012). Empowering leaders optimize working conditions for engagement: a multilevel study. *J. Occup. Health Psychol.* 17 15–27. 10.1037/a0025942 22409390

[B38] Van den Heuvel M. Demerouti E. Peeters M. C. W. (2015). The job crafting intervention: effects on job resources, self-efficacy, and affective well-being. *J. Occup. Organ. Psychol.* 88 511–532. 10.1111/joop.12128

[B39] Van Wingerden J. (2016). *Job Demands-Resources Interventions.* Doctoral dissertation, Erasmus University Rotterdam, Rotterdam 10.1108/JMP-03-2014-0086

[B40] Van Wingerden J. Bakker A. B. Derks D. (2015). The impact of personal resources and job crafting interventions on work engagement and performance. *Hum. Resour. Manage.* 56 51–67. 10.1002/hrm.21758

[B41] Van Wingerden J. Bakker A. B. Derks D. (2017a). Fostering employee well-being via a job crafting intervention. *J. Vocat. Behav.* 100 164–174. 10.1016/j.jvb.2017.03.008

[B42] Van Wingerden J. Bakker A. B. Derks D. (2017b). The longitudinal impact of a job crafting intervention. *Eur. J. Work Organ. Psychol.* 26 107–119. 10.1080/1359432X.2016.1224233

[B43] Van Wingerden J. Derks D. Bakker A. B. Dorenbosch L. (2013). Job crafting in het speciaal onderwijs: een kwalitatieve analyse [Job crafting in special education: a qualitative analysis]. *Gedrag Organisatie* 26 85–103.

[B44] Van Wingerden J. Niks I. (2017). Construction and validation of the perceived opportunity to craft scale. *Front. Psychol.* 8:573. 10.3389/fpsyg.2017.00573 28446893 PMC5388759

[B45] Williams L. J. Anderson S. E. (1991). Job satisfaction and organizational commitment as predictors of organizational citizenship and in-role behaviors. *J. Manage.* 17 601–617. 10.1177/014920639101700305

[B46] World Economic Forum (2017). *Realizing Human Potential in the Fourth Industrial Revolution: An Agenda for Leaders to Shape the Future of Education, Gender and Work.* Geneva: World Economic Forum.

[B47] Wrzesniewski A. (2003). “Finding positive meaning in work,” in *Positive Organizational Scholarship: Foundations of a New Discipline*, eds Cameron K. S. Dutton J. E. Quinn R. E. (San Francisco, CA: Berrett-Koehler), 298–308.

[B48] Wrzesniewski A. Dutton J. E. (2001). Crafting a job: revisioning employees as active crafters of their work. *Acad. Manage. Rev.* 26 179–201.

